# Associations of self-compassion and resilience with help-seeking among Chinese athletes: the mediating role of self-stigma and sex differences

**DOI:** 10.3389/fpsyg.2026.1776657

**Published:** 2026-03-27

**Authors:** Yan Liu, Ziyou Huang, Yajie Xu, Jihan Wang

**Affiliations:** School of Physical Education, Anqing Normal University, Anqing, China

**Keywords:** behavioral intention to help-seeking, Chinese athletes, personal copingresources, resilience, self-compassion, self-stigma of help-seeking, sex differences

## Abstract

**Introduction:**

Mental health problems are prevalent among athletes. However, the utilization of professional psychological services remains remarkably low. Although behavioral intention to help-seeking (BI-HS) is a critical precursor to actual help-seeking behavior and serves as a protective factor for mental health, little is known about how personal coping resources are associated with BI-HS among athletes, nor about the potential psychological mechanisms underlying these associations. The present study examined the associations between personal coping resources (self-compassion and resilience) and BI-HS, and further tested the mediating role of self-stigma of help-seeking (SSHS) in these relationships. Sex differences in the proposed model were also investigated.

**Methods:**

A total of 684 Chinese athletes participated in the study. Structural equation modeling (SEM) and multi-group SEM analyses were conducted.

**Results:**

The findings indicated that both self-compassion and resilience were positively associated with BI-HS. SEM analyses further demonstrated that SSHS significantly mediated the relationships between self-compassion and BI-HS, as well as between resilience and BI-HS. Moreover, multi-group SEM analyses revealed a significant sex difference: the effect of SSHS on BI-HS was significant among male athletes but not among female athletes.

**Conclusions:**

These findings suggest that personal coping resources may enhance athletes' BI-HS partly by reducing self-stigma, with this mechanism being particularly salient for male athletes. Interventions aimed at fostering self-compassion and resilience while addressing SSHS may be especially effective in promoting BI-HS among athletes, especially males.

## 1 Introduction

Athletes face substantial psychological demands, including engagement, performance, interpersonal relationships, and evaluation from coaches and peers (Ziyou et al., 2024; Bentzen et al., 2025). These stressors may place athletes at higher risk of mental distress (Nuetzel, 2025). Evidence suggests a notable burden of mental health problems in athlete populations. A meta-analysis across 22 studies reported a pooled prevalence of mental health disorder of 34.0% among athletes (Gouttebarge et al., 2019). Another meta-analysis of former elite athletes found anxiety and depression were more than twice as prevalent as in the general population (Runacres and Marshall, 2024). When early symptoms are not addressed, even mild anxiety or depressive symptoms can progress into anxiety disorders or major depressive disorder (World Health Organization, 2021). Such escalation may lead to severe outcomes, including non-suicidal self-injury and suicide (Huang et al., 2025a). These highlight the importance of early identification and intervention for mental health problems in athletes.

Seeking professional help is widely regarded as an effective strategy to reduce mental health problems and prevent symptom deterioration (Rickwood et al., 2005). Nevertheless, athletes often underutilize mental health services, even when experiencing significant distress (McCabe et al., 2023). A recent systematic review and meta-analysis reported that only 22.4% of athletes engaged in help-seeking (Cosh et al., 2024). This low rate is concerning given the high prevalence of mental health problems in this population. Understanding factors that influence help-seeking is therefore critical. Help-seeking behavior is typically preceded by behavioral intention to help-seeking (BI-HS), which represents a preparatory stage and a key predictor of subsequent action (Lu et al., 2024). In the present study, BI-HS refers to intentions to seek support from psychiatrists or counselors (Lu et al., 2025).

According to Stress Coping Theory, coping resources influence coping responses (Lazarus and Folkman, 1984). In the context of mental health, BI-HS represents a constructive coping response to psychological distress. Self-compassion is a personal coping resource and refers to an attitude of kindness and understanding toward one's own suffering and personal shortcomings (Neff, 2003). Individuals with higher self-compassion tend to respond to distress with care rather than self-judgment (Schaab et al., 2024). This response style may reduce shame and self-criticism, allowing individuals to acknowledge mental health difficulties without excessive defensiveness or avoidance of professional support (Neff, 2023). Such acceptance may reduce defensive reactions and avoidance of professional support. Although direct evidence linking self-compassion to BI-HS in athletes remains limited, existing research provides relevant support. Studies in athlete populations indicate that higher self-compassion is associated with more adaptive emotional regulation and reduced avoidance in response to stress and failure (Doorley et al., 2022; Kuchar et al., 2023). In addition, self-compassion in collegiate athletes predicts faster recovery from negative emotional experiences, suggesting greater openness to addressing distress constructively (Zhang et al., 2023). Evidence from college student samples further indicates that self-compassion is associated with health-promoting motivations (Brenner et al., 2018) and professional help-seeking (Heath et al., 2017, 2018). Empirical studies suggest that individuals with higher self-compassion are more willing to seek help for mental health difficulties (Ishikawa et al., 2023). These findings suggest that self-compassion may facilitate athlete BI-HS.

Resilience is another key personal coping resource and is commonly defined as the ability to adapt, persist, and solve problems in the face of adversity (Farchi and Peled-Avram, 2025). Within sport psychology, resilience has been extensively examined as a central protective factor in high-performance contexts (Stoyanova et al., 2025). Sport-specific models conceptualize resilience as the dynamic interaction of personal assets, environmental resources, and cognitive processes that enable athletes to maintain or regain functioning under sport-related stressors such as injury, performance pressure, and competitive failure (Fletcher and Sarkar, 2012; Fonseca Bicalho et al., 2021). Recent reviews further emphasize that resilience in athletes is shaped by sport-specific stressors and protective mechanisms, including cognitive appraisal, social support, and adaptive coping, underscoring its critical role in safeguarding mental well-being and sustaining performance (Gupta and McCarthy, 2022). In parallel, empirical research on resilience in sport has expanded rapidly, further consolidating its status as a core psychological resource in athletic populations (Fonseca Bicalho et al., 2021).

Empirical research consistently indicates that resilience is associated with greater engagement in health-promoting behaviors (Chrétien et al., 2024). For instance, a recent study of Chinese student-athletes found that resilience was positively correlated with BI-HS among college athletes (Liao et al., 2024). Additionally, evidence from research on student-athletes shows that resilience relates to help-seeking behavior in the context of depressive and anxiety symptoms (Lee et al., 2025). Moreover, resilience training programs implemented in college athletes have been shown to enhance adaptive coping strategies that may facilitate help-seeking when facing stressors (Sullivan et al., 2023). These findings suggest that resilience, particular within sport-specific contexts, may facilitate BI-HS by promoting flexible appraisal, resource mobilization, and proactive problem-solving in response to mental health challenges among athletes.

Self-stigma of help-seeking (SSHS) refers to the fear that seeking professional support will diminish one's self-regard, self-confidence, and perceived personal worth (Vogel et al., 2006). Individuals experiencing high SSHS may interpret seeking help as a sign of personal weakness, which undermines their willingness to seek psychological services (Schomerus et al., 2019). Previous research has consistently shown that SSHS is negatively associated with BI-HS (Ojio et al., 2021; Hilliard et al., 2022; Bu et al., 2024). Thus, SSHS represents a critical psychological barrier that may explain why individuals avoid seeking help despite experiencing mental health difficulties. Personal coping resources may influence BI-HS indirectly through SSHS. Individuals with higher self-compassion tend to respond to distress with understanding rather than self-criticism, which may reduce fears of self-worth loss associated with BI-HS (Biber and Ellis, 2019). Empirical studies indicate that self-compassion is associated with lower SSHS and more favorable attitudes toward BI-HS (Heath et al., 2018). Similarly, resilience may buffer against self-stigmatizing beliefs by fostering adaptive appraisals of stress and greater confidence in coping with challenges (Bu et al., 2024). Resilient individuals may be less likely to view help-seeking as a personal failure. Reduced self-stigma, in turn, may facilitate stronger BI-HS. Therefore, SSHS may serve as a key mediator linking self-compassion and resilience to BI-HS among athletes.

Sex differences may play an important role in understanding BI-HS among athletes. Prior research shows that males are generally less willing to seek psychological support compared with female (Güney et al., 2024; Usenik and Kranjec, 2025). Consistent with this pattern, recent evidence suggests that female athletes show higher BI-HS regarding mental health problems than their male counterparts (Bird and Earl, 2026). Large-scale reviews further demonstrate that male and female athletes differ in mental health-related attitudes and behaviors, highlighting the importance of considering sex when examining help-seeking processes in sport contexts (Cosh et al., 2024; Kew et al., 2024; Walton et al., 2024). These differences may stem from gender-related norms that encourage emotional control, self-reliance, and the avoidance of behavior perceived as reflecting weakness (Addis and Mahalik, 2003; Ojio et al., 2025). Such norms can increase SSHS, particularly among males. Studies also indicate that males report greater fears of self-worth loss when considering professional psychological services, which may further reduce their BI-HS (Sheikh et al., 2025). In contrast, female athletes tend to express more openness toward psychological support and report lower levels of SSHS (Cosh et al., 2024). Sex differences may also emerge in psychological resources. Research suggests that females often report higher self-compassion, whereas males may report greater reluctance to acknowledge distress (Keyes et al., 2022). These differences may shape how self-compassion and resilience influence SSHS and BI-HS. Accordingly, examining sex-specific pathways is necessary for understanding variability in BI-HS and for developing targeted interventions in athletic populations.

Despite growing research on athletes' mental health and help-seeking, several important gaps remain. First, most existing studies have focused on external barriers, such as service accessibility and social support (Gulliver et al., 2012), while relatively little attention has been paid to the role of personal coping resources (e.g., self-compassion and resilience) in shaping BI-HS. Second, although self-compassion and resilience have been examined separately in relation to mental health outcomes (Chuang et al., 2024), their joint effects and underlying psychological mechanisms on BI-HS among athletes are still poorly understood. Third, empirical evidence among Chinese athletes remains limited, particularly regarding how personal coping resources influence BI-HS. Finally, little is known about whether the mechanisms linking coping resources to BI-HS differ by sex in athletic populations. Addressing these gaps is essential for developing more effective and evidence-based mental health interventions for athletes.

Given the background, the present study investigated the prevalence of BI-HS among athletes and the associations between personal coping resources (self-compassion and resilience) and BI-HS. It also tested the mediation effect of SSHS between the two types of personal coping resources and BI-HS. The following hypotheses were proposed: (H1) Self-compassion would be positively associated with BI-HS; (H2) Resilience would be positively associated with BI-HS; (H3) SSHS would mediate the association between self-compassion and BI-HS; (H4) SSHS would mediate the association between resilience and BI-HS; (H5) The associations between self-compassion, resilience, SSHS, and BI-HS would differ by sex.

## 2 Methods

### 2.1 Participants and data collection

Participants were Chinese athletes recruited from the sports college of university athletic teams in Anhui Province, China. About 75% of the sample consisted of student-athletes who were enrolled in full-time academic programs while simultaneously engaging in regular training and competition, whereas the remaining participants were full-time professional athletes. Eligible participants were adult athletes (aged 18 years or above) who were currently engaged in regular athletic training. Individuals who were unable to complete the questionnaire independently or who provided incomplete responses were excluded from the analysis. Data were collected between March and June 2025 using a structured, self-administered questionnaire. Trained research staff collaborated with coaches and administrative personnel to distribute and collect the surveys on site. Prior to participation, all athletes received an explanation of the study's purpose, procedures, and confidentiality measures. Written informed consent was obtained from all participants. This study was approved by the research ethics committee of the corresponding author's affiliated institution (Approval number: AQNU2025211).

A total of 708 questionnaires were distributed, and 684 valid responses were retained after data screening, resulting in a response rate of 96.6%. The final sample consisted of 684 athletes, 63.5% were male and 36.5% were female. 50.3% were involved in team sports, while the remainder participated in individual, weight-class, or aesthetic sports. 61.7% training more than 16 hours per week, and the majority had received college-level education.

### 2.2 Measures

#### 2.2.1 Background factors

Background information was collected on age, years of training, sex, educational level, perceived family financial situation, type of sport, weekly training hours, and previous help-seeking experience.

#### 2.2.2 BI-HS

BI-HS was assessed using a single-item measure extracted from the Intentions to Seek Counseling Inventory (ISCI; Cash et al., 1975; Kelly and Achter, 1995; Kim and Zane, 2016), which was used to assess participants' intention to seek help from professionals if they were experiencing specific mental health problems. This measure has been used in prior studies conducted in Chinese samples (Lu et al., 2024, 2025; Sun et al., 2026). Participants were asked, “In the case of having mental health problems (e.g., anxiety or depressive symptoms), how likely would you seek help from a psychiatrist or a counselor (yes or no response)?”

#### 2.2.3 Self-compassion

Self-compassion was assessed by using the 12-item short form of the Self-Compassion Scale (Raes et al., 2011). Sample items include “When I fail at something important to me, I become consumed by feelings of inadequacy” and “When I'm feeling down, I tend to feel like most other people are probably happier than I am”. All items are rated with a 5-point Likert scale (1 = almost never to 5 = almost always), with a higher score indicating higher levels of self-compassion. It has been validated in Chinese adults and showed excellent psychometric properties (Meng et al., 2019). In the present study, the Cronbach's alpha of the scale was 0.87.

#### 2.2.4 Resilience

Resilience was measured using the 2-item abbreviated Connor–Davidson Resilience Scale (CD-RISC-2; Vaishnavi et al., 2007). This scale has been validated in Chinese population and demonstrated satisfactory psychometric properties (Ni et al., 2016). In addition, this scale has been widely applied in previous studies conducted in Chinese samples across different contexts, supporting its applicability and robustness (Lai et al., 2020; Hou et al., 2021; Zhang et al., 2021). The two items are “Able to adapt to change” and “Tend to bounce back after illness or hardship,” rated on a 5-point scale (0 = not true at all to 4 = true nearly all the time), with a higher score indicating higher levels of resilience. In the present study, the Cronbach's alpha coefficient for the scale was 0.83.

#### 2.2.5 Self-stigma of help-seeking (SSHS)

SSHS was assessed using the 10-item Self-Stigma of Seeking Help (SSOSH) scale (Vogel et al., 2006). Sample items include “It would make me feel inferior to ask a therapist for help” and “I would feel inadequate if I went to a therapist for psychological help.” All items are rated on a 5-point Likert scale (1 = strongly disagree to 5 = strongly agree), with higher scores indicating greater levels of self-stigma toward seeking psychological help. The Chinese version of the scale has demonstrated good reliability and validity in prior research (Huang et al., 2025b). In the present study, the Cronbach's alpha of the scale was 0.93.

### 2.3 Data analysis

Descriptive statistics were first summarized. Univariate logistic regression was applied to test the relationships between background factors and BI-PHSMHP. Pearson correlation coefficients were computed to assess associations among continuous variables, whereas Spearman correlations were used for examining relationships between the main study variables and BI-HS. Multiple logistic regression analyses, adjusting for background factors that showed significant univariate associations, were then conducted to evaluate the associations between each key predictor and BI-HS.

Structural equation modeling (SEM) was further employed to test whether SSHS mediated the associations of self-compassion and resilience with BI-HS. Background variables that demonstrated significant associations with BI-HS were controlled as covariates in the SEM. The weighted least square mean and variance adjusted (WLSMV) estimator was used for model estimation. To further examine potential sex differences in the mediation model, a multi-group SEM across sex was conducted, in which model parameters were compared between male and female participants. Satisfactory model fit indices included Comparative Fit Index (CFI) ≥ 0.90, Tucker-Lewis Index (TLI) ≥ 0.90, Root Mean Square Error of Approximation (RMSEA) ≤ 0.08, and Standardized Root Mean Square Residual (SRMR) ≤ 0.08 (Kline, 2016). All SEM analyses were conducted by using Mplus 8.3 while the other analyses by SPSS 23.0. Statistical significance was defined as a two-tailed *p* < 0.05.

## 3 Results

### 3.1 Descriptive analysis

The athletes had a mean age of 21.2 years (SD = 2.3) and an average training years of 11.1 years (SD = 2.0). Men accounted for 63.5% of the sample. Approximately three-quarters of the participants had a university-level education (75.1%), and 69.6% reported a middle-level family income. Half of the athletes participated in team sports (50.3%), while about one-quarter were engaged in individual sports (25.7%). Most participants trained more than 16 hours per week (61.7%). In total, 14.3% had previously sought psychological help, and 17.5% showed BI-HS (Table 1).

**Table 1:** Descriptive analyses of background factors and its associations with BI-HS.

**Continuous variable**	**Mean**	**SD**	**ORu**	**95% CI**
Age	21.2	2.3	1.15 ^**^	(1.06, 1.24)
Years of training	11.1	2.0	1.15 ^**^	(1.06, 1.26)
**Categorical variable**	**n**	**%**		
**Sex**				
Male	434	63.5	Ref	
Female	250	36.5	2.21 ^***^	(1.48, 3.31)
**Educational level**				
High school or equal	60	8.8	Ref	
College	514	75.1	2.13	(0.89, 5.09)
College above	110	16.1	1.31	(0.47, 3.61)
**Perceived family financial situation**				
Below average	164	24.0	Ref	
Average	476	69.6	1.09	(0.66, 1.80)
Above average	44	6.4	3.98 ^***^	(1.90, 8.35)
**Type of sport**				
Team sports	344	50.3	Ref	
Individual sports	176	25.7	1.04	(0.65, 1.68)
Weight-class sports	68	9.9	0.81	(0.39, 1.68)
Aesthetic sports	96	14.0	0.94	(0.51, 1.72)
**Weekly training hours**				
< 5h	74	10.8	Ref	
5-10h	60	8.8	4.12 ^**^	(1.50, 11.34)
11-15h	128	18.7	0.76	(0.25, 2.27)
16-20h	222	32.5	2.34	(0.95, 5.79)
> 20h	200	29.2	3.83 ^**^	(1.57, 9.39)
**Previous help-seeking experiences**				
Yes	98	14.3	Ref	
No	586	85.7	1.40 ^***^	(1.25, 1.65)
**BI-HS**				
No	564	82.5	N.A	N.A
Yes	120	17.5	N.A	N.A

ORu = univariate odds ratio; N.A = not applicable. SSHS = self-stigma with help-seeking; BI-HS = behavioral intention to help-seeking. ^**^p < 0.01, ^***^p < 0.001.

### 3.2 Background factors of BI-HS

Older age (ORu = 1.15) and longer weekly training hours (ORu = 1.15) were associated with greater likelihood of BI-HS. Compared with men, women were more likely to exhibit BI-HS (ORu = 2.21). Relative to those with below-average income, athletes with above-average income showed stronger BI-HS (ORu = 3.98). Compared with athletes training fewer than 5 hours per week, those training 5–10 hours (ORu = 4.12) or more than 20 hours (ORu = 3.83) were more likely to display BI-HS. Athletes who had previously sought help were also more inclined to BI-HS (ORu = 1.40). Educational level and type of sport were not significantly associated with BI-HS (Table 1).

### 3.3 Key study variables of BI-HS

As shown in Table 2, self-compassion (ORa = 1.35) and resilience (ORa = 1.62) were positively associated with BI-HS. In contrast, SSHS was negatively associated with BI-HS (ORa = 0.92).

**Table 2:** Associations between main variables and BI-HS.

**Variable**	**ORu**	**95% CI**	**ORa**	**95% CI**
Self-compassion	1.41 ^***^	(1.22, 1.63)	1.35 ^***^	(1.16, 1.56)
Resilience	1.29 ^***^	(1.13, 1.48)	1.62 ^***^	(1.36, 1.92)
SSHS	0.93 ^***^	(0.90, 0.96)	0.92 ^***^	(0.88, 0.96)

ORu = univariate odds ratio; ORa = adjusted odds ratio. SSHS = self-stigma with help-seeking; BI-HS = behavioral intention to help-seeking. ^***^p < 0.001.

### 3.4 Correlations

As presented in Table 3, self-compassion, resilience, and BI-HS were positively correlated with each other (*r*: 0.20–0.56). By contrast, self-compassion, resilience, and BI-HS were negatively SSHS (*r*: −0.49–−0.24).

**Table 3:** Mean, SD, and correlations.

**Variable**	**1**	**2**	**3**	**4**
Self-compassion	1			
Resilience	0.56^***^	1		
SSHS	−0.41^***^	−0.49^***^	1	
BI-HS	#0.20^***^	#0.24^***^	#-0.24^***^	1
Mean	33.10	7.82	31.05	3.27
SD	9.54	1.59	9.70	1.26

#Spearman correlations were conducted for these variables, while Pearson correlations were used for all other variables. ^***^p < 0.001. SSHS = self-stigma with help-seeking; BI-HS = behavioral intention to help-seeking.

### 3.5 SEM

**Figure 1:**
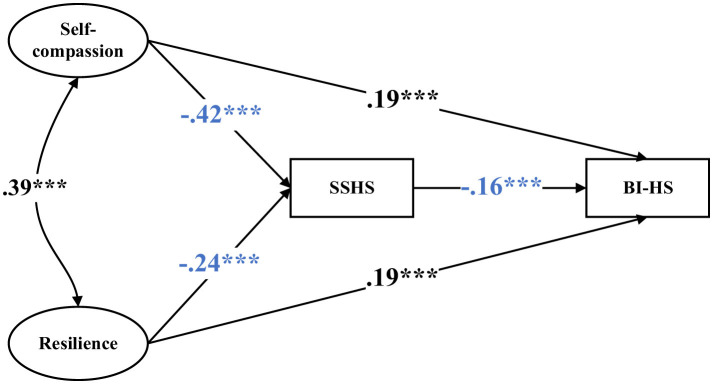
Structural equation modeling. Standardized coefficients are reported. ^***^, *p* < 0.001. SSHS = self-stigma of help-seeking; BI-HS = behavioral intention to help-seeking. The model was adjusted for age, years of training, perceived family financial situation, weekly training hours, and previous help-seeking experience.

Figure 1 shows the results of the SEM. The SEM yielded satisfactory model fit indices (CFI = 0.991, TLI = 0.987, RMSEA = 0.023, SRMR = 0.035). Self-compassion was negatively associated with SSHS (β = −0.42, *p* < 0.001), and SSHS, in turn, was negatively associated with BI-HS (β = −0.16, *p* < 0.001). Self-compassion also showed a significant direct association with BI-HS (β = 0.19, *p* < 0.001). These findings indicate that SSHS partially mediated the relationship between self-compassion and BI-HS. Similarly, resilience was negatively associated with SSHS (β = −0.24, *p* < 0.001), and SSHS was again negatively associated with BI-HS (β = −0.16, *p* < 0.001). Resilience was also directly and positively related to BI-HS (β = 0.19, *p* < 0.001). Thus, SSHS also served as a partial mediator between resilience and BI-HS.

### 3.6 Multi-group SEM

A multi-group SEM was conducted to examine whether the structural paths differed between male and female athletes. The unconstrained model, in which all structural paths were freely estimated across groups, demonstrated good model fit (CFI = 0.981; TLI = 0.974; RMSEA = 0.037; SRMR = 0.045). The constrained model, in which all structural paths were set to be equal across sex, showed a significantly poorer fit (CFI = 0.965; TLI = 0.960; RMSEA = 0.057; SRMR = 0.054). Comparison of model fit indices indicated a meaningful decrement in fit (ΔCFI > 0.01; ΔRMSEA > 0.015), suggesting that the structural path differed between males and females.

**Figure 2:**
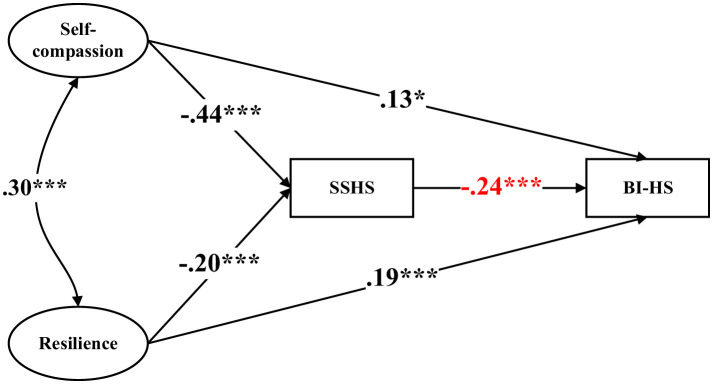
Structural equation modeling among males. Standardized coefficients are reported. ^*^, *p* < 0.05, ^***^, *p* < 0.001. SSHS = self-stigma of help-seeking; BI-HS = behavioral intention to help-seeking. The model was adjusted for age, years of training, perceived family financial situation, weekly training hours, and previous help-seeking experience.

**Figure 3:**
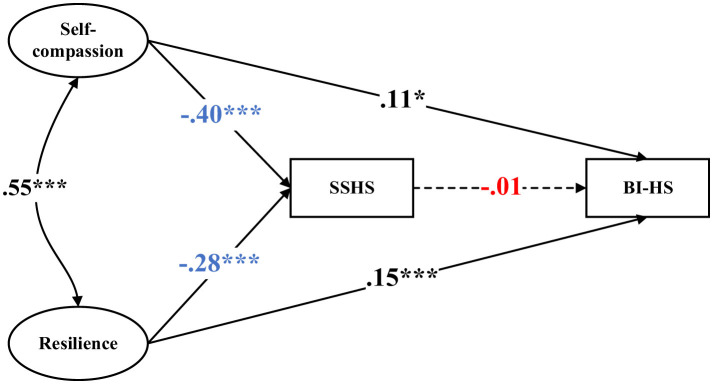
Structural equation modeling among females. Standardized coefficients are reported. ^*^, *p* < 0.05, ^***^, *p* < 0.001. SSHS = self-stigma of help-seeking; BI-HS = behavioral intention to help-seeking. The model was adjusted for age, years of training, perceived family financial situation, weekly training hours, and previous help-seeking experience.

Further examination of individual path constraints revealed that only the path from SSHS to BI-HS significantly varied by sex (Figure 2 and Figure 3). For male athletes, SSHS was negatively associated with BI-HS (β = −0.24, *p* < 0.001), whereas this association was not statistically significant among female athletes. All other structural paths were statistically equivalent across sex groups.

## 4 Discussion

The present study investigated the prevalence of BI-HS and the associations between self-compassion/resilience and BI-HS among Chinese athletes. We further tested whether SSHS mediates these associations and whether the structural paths differ by sex. The findings revealed a low prevalence of BI-HS of 17.5% in this athletic population. Both self-compassion and resilience were positively associated with BI-HS. SSHS significantly mediated the association between self-compassion/resilience and BI-HS. Additionally, the effect of SSHS on BI-HS was significant for male but not for female athletes. These results highlight the importance of considering internal psychological resources, internalized stigma, and sex-specific norms when attempting to understand and promote BI-HS among athletes.

### 4.1 Prevalence of BI-HS among athletes

The prevalence of BI-HS in this sample was 17.5%, indicating that fewer than one in five athletes expressed a clear intention to seek professional help. Although this rate appears low, it is broadly consistent with previous research. For example, recent meta-analytic evidence indicates that approximately 22.4% of athletes report engaging in mental health help-seeking behaviors, which is comparable to the prevalence observed in the present study (Cosh et al., 2024). In addition, a study among Chinese adolescents found that the prevalence of intention to seek professional help was 15.4%, suggesting that low levels of help-seeking intention may also be common in Chinese youth populations (Lu et al., 2024).

This low rate is striking given the high psychological demands of competitive sport and the growing recognition of athlete mental health. Athletes often train and compete in environments that prioritize performance, endurance, and mental toughness (Walton et al., 2024). Within such settings, admitting psychological difficulties or considering formal psychological support may be seen as inconsistent with expectations of strength and self-control (Ojio et al., 2025). These norms can create substantial personal and interpersonal costs for acknowledging distress and contemplating professional help (Purcell et al., 2022). Consequently, athletes may experience a pronounced gap between psychological need and actual willingness to engage with professional mental health services.

It should also be noted that BI-HS was assessed using a single-item indicator in the present study, which may limit measurement precision and potentially influence prevalence estimates. Therefore, although the observed rate is consistent with existing evidence, future studies using comprehensive multi-item measures are needed to further validate the prevalence of help-seeking intention in athletic populations. Nevertheless, the convergence between the present findings and prior research suggests that low BI-HS represents a substantive concern rather than a mere measurement artifact. This gap highlights the necessity of interventions that directly address help-seeking intentions and the perceived meanings of professional support, rather than assuming that psychological need will automatically translate into help-seeking behaviors.

### 4.2 Background factors and BI-HS

Several socio-demographic factors were associated with BI-HS in the present study. Older age and longer weekly training hours were linked to a higher likelihood of reporting BI-HS. This pattern may reflect greater cumulative exposure to physical and psychological stressors over time, which may increase athletes' awareness of mental health needs and their perceived benefits of professional support (McLoughlin et al., 2021). Athletes with heavier training loads may also experience more pronounced fatigue, performance pressure, and injury risk, making external psychological resources more salient (Purcell et al., 2019). Female athletes were more likely to report BI-HS than male athletes, which is consistent with previous findings showing greater openness toward psychological support among females (Bird and Earl, 2026). In addition, athletes with higher perceived family financial status showed stronger BI-HS, possibly because financial security reduces concerns about the cost, accessibility, and potential long-term consequences of seeking professional services (Shi et al., 2020).

Previous help-seeking experience was also positively associated with BI-HS, suggesting that earlier contact with mental health professionals may reduce uncertainty and fear related to counseling, thus lowering psychological barriers to future help-seeking (Sun et al., 2026). Familiarity with service processes and positive prior experiences may promote more favorable attitudes toward professional support. In contrast, educational level and type of sport were not significantly related to BI-HS in this sample. This finding may indicate that help-seeking intentions are shaped more strongly by personal psychological resources and perceived barriers than by formal educational attainment or sport-specific characteristics. Alternatively, the relatively homogeneous educational background and training environment of the present sample may have limited variability in these factors, thereby reducing their explanatory power.

### 4.3 Associations of self-compassion and resilience with BI-HS

In line with H1, our results demonstrate a positive association between self-compassion and BI-HS. Athletes who were more self-compassionate were more likely to report an intention to seek professional help. This association can be understood through the way self-compassion alters one's relationship with personal failure and emotional pain. When athletes respond to setbacks and distress with understanding rather than harsh self-judgment, they may be less inclined to interpret psychological difficulties as evidence of personal defect (Muris and Otgaar, 2023). In turn, the idea of seeking help becomes more compatible with maintaining self-worth and a coherent performance identity (Kuchar et al., 2023). In performance-focused sport environments, where athletes may feel pressure to appear strong and in control, self-compassion provides internal permission to acknowledge suffering without equating it with inadequacy (Neff, 2023). This internal stance makes it more acceptable to view professional help as a legitimate and caring response to one's own struggles, rather than as a sign of weakness (Hilliard et al., 2019).

Consistent with H2, the present findings indicate that resilience was also positively associated with BI-HS. At first glance, this may appear counterintuitive if resilience is narrowly equated with “enduring alone.” However, resilience is better conceptualized as the capacity to adapt flexibly and to use effective strategies when facing adversity (Wu et al., 2013). Athletes with higher resilience may be more likely to view stressors as manageable challenges rather than as catastrophic threats (Sarkar and Fletcher, 2014). Within this mindset, seeking professional help can be incorporated as a proactive, problem-focused strategy rather than as a sign of surrender. In other words, resilient athletes may see drawing on external support as part of taking responsible action to cope with difficulties and optimize performance (Sarkar and Page, 2022; Cao et al., 2024). This interpretation challenges the simplistic notion that “the more resilient you are, the less you need help” and instead suggests that genuine resilience may include the willingness to mobilize appropriate resources, including professional psychological services (Olsson and Skoog, 2025).

### 4.4 The mediating role of SSHS

H3 was supported, as SSHS significantly mediated the association between self-compassion and BI-HS. The mediating role of SSHS sheds light on the mechanism through which self-compassion influences BI-HS. Self-compassion involves recognizing distress while maintaining a kind and balanced view of oneself. This stance may weaken the belief that needing help implies being weak, defective, or unworthy (Min et al., 2022). When athletes hold fewer self-stigmatizing beliefs about help-seeking, they are less likely to anticipate a loss of self-worth if they consult a mental health professional (Vogel et al., 2006). In this way, self-compassion does not only make distress more bearable; it also reshapes the anticipated meaning of seeking help (Hilliard et al., 2019). BI-HS can be reinterpreted as an act of self-care rather than self-betrayal. The mediation via SSHS suggests that a key pathway from self-compassion to BI-HS lies in reducing fears that professional support will damage one's self-image, an issue that is especially salient in cultures where reputation and strength are highly valued.

H4 was supported, as SSHS also significantly mediated the relationship between resilience and BI-HS. The mechanism here appears to be somewhat different from that of self-compassion but complementary. Resilience is closely tied to a sense of efficacy in dealing with adversity and to a belief that obstacles can be managed with appropriate strategies (Schwarzer, 2024). When athletes feel confident in their ability to confront and navigate difficulties, they may be more inclined to frame professional help as an additional resource that can be integrated into their coping repertoire (Crowe et al., 2016). Under this interpretation, help-seeking is not seen as relinquishing control but as expanding the toolkit for managing challenges. Such a view naturally reduces self-stigmatizing beliefs that equate help-seeking with personal failure (Sum et al., 2024). Thus, resilience may influence BI-HS partly by transforming help-seeking from a symbol of defeat into a rational, agentic choice in the face of stress (Lu et al., 2025). This highlights that resilience, when understood as flexible and resource-oriented coping, can work against internalized messages that “real athletes should handle everything on their own.

### 4.5 Sex differences

The present study found that the effect of SSHS on BI-HS was significant for male but not for female athletes, partly supporting H5. Specifically, the path from SSHS to BI-HS was significantly negative among male athletes but non-significant among female athletes, suggesting that SSHS operates as a more critical barrier to BI-HS for males. One plausible explanation is that gender norms are most strongly activated at the level of overt help-seeking decisions. Male athletes may interpret the act of seeking professional help as a direct violation of expectations of strength and self-reliance, so once self-stigmatizing beliefs are present, they translate more directly into reduced help-seeking intention (Ojio et al., 2025). In contrast, female athletes may still hold some stigmatizing beliefs, but their decisions about help-seeking may be more strongly influenced by other considerations, such as emotional need, perceived benefits of counseling, or support from significant others (Huang et al., 2025c), which weakens the direct impact of SSHS on BI-HS. The absence of sex differences in the other paths suggests that self-compassion and resilience relate to SSHS and BI-HS in broadly similar ways for male and female athletes. In other words, both sexes seem to draw on these psychological resources in comparable ways, but the point at which SSHS is converted into a decision about whether to seek help is where gender norms exert their strongest and most differentiated influence.

### 4.6 Practical implications and Limitations

These findings have several practical implications. First, the low prevalence of BI-HS suggests that athlete mental health initiatives should explicitly target help-seeking intentions, rather than assuming that symptom education alone is sufficient. Screening, psycho education, and routine mental health check-ins within teams could incorporate discussions about when and how to seek professional support. Second, interventions that cultivate self-compassion and resilience may be promising, as they appear to encourage help-seeking intention while simultaneously challenging negative self-judgments about needing assistance. However, such interventions should be designed to frame help-seeking as consistent with strength and professionalism, particularly for male athletes. Third, stigma reduction needs to be integrated into mental health promotion. For male athletes, this might involve directly contesting the idea that consulting a psychologist is incompatible with being tough or committed, using coaches and respected peers as role models. For female athletes, efforts may focus more on improving access, ensuring confidentiality, and normalizing conversations about mental health within team environments.

Despite these strengths, several limitations should be considered. First, the cross-sectional design prevents causal conclusions. Future studies should use longitudinal or experimental designs to clarify temporal and causal relationships. Second, all data were collected via self-report, raising concerns about social desirability. Future research should incorporate more objective indicators, such as behavioral data, implicit measures, or peer and coach reports. Third, the sample consisted of Chinese athletes from one province, which may limit generalizability to other cultural contexts or non-athlete groups. Future studies should replicate these models in different countries, sport systems, and more diverse populations. Fourth, BI-HS reflects intention rather than actual help-seeking behavior and was assessed using a single-item indicator, which may limit measurement precision. Future studies should employ multi-item measures and longitudinal designs to examine whether intentions translate into actual help-seeking. Fifth, resilience was measured using the abbreviated CD-RISC-2. Although validated in Chinese samples, brief measures may not capture all sport-specific aspects of resilience. Future studies are encouraged to use longer or sport-specific instruments where appropriate. Finally, SSHS accounted for only part of the associations between self-compassion, resilience, and BI-HS. Future research should test additional mediators, such as mental health literacy, perceived need, and coach or teammate norms.

## 5 Conclusions

This study examined associations between self-compassion, resilience, SSHS, and BI-HS among Chinese athletes. The prevalence of BI-HS was low, indicating a substantial gap between potential psychological need and willingness to seek professional support. Higher self-compassion and resilience were associated with greater BI-HS, partly through lower SSHS, suggesting that psychological resources can reshape beliefs about help-seeking. SSHS predicted BI-HS only among male athletes, indicating that gendered norms make self-stigma a particularly important barrier for men. In contrast, female athletes' SSHS may depend more on other factors, such as emotional needs or perceived benefits of counseling. Overall, the findings highlight self-compassion, resilience, and self-stigma as key targets for promoting SSHS in athletic settings.

## Data Availability

The original contributions presented in the study are included in the article/supplementary material, further inquiries can be directed to the corresponding author/s.
